# Transcreating approaches to addressing disproportionate impacts on health in Colorado communities: the Colorado Community Engagement Alliance multi-year study

**DOI:** 10.3389/fpubh.2026.1728986

**Published:** 2026-02-12

**Authors:** Meredith K. Warman, Ricardo Gonzalez-Fisher, Charlene Barrientos Ortiz, Jerica M. Berge, Douglas H. Fernald, Meredith P. Fort, Zachary Giano, Susan L. Moore, Montelle Tamez, Linda Zittleman, Donald E. Nease

**Affiliations:** 1 Department of Family Medicine, School of Medicine, University of Colorado Anschutz Medical Campus, Aurora, CO, United States; 2 Servicios de la Raza, Denver, CO, United States; 3 Colorado Clinical and Translational Sciences Institute, University of Colorado Anschutz Medical Campus, Aurora, CO, United States; 4 Department of Health Systems, Management and Policy, Colorado School of Public Health, University of Colorado Anschutz Medical Campus, Aurora, CO, United States; 5 Adult & Child Center for Outcomes Research & Delivery Science, University of Colorado Anschutz Medical Campus, Aurora, CO, United States

**Keywords:** Boot Camp Translation, chronic disease, community-engaged research, systems thinking, transcreation

## Abstract

**Background:**

Social and structural factors that influence health continue to affect communities across Colorado, with disproportionate impact in populations that have historically experienced limited access to resources and opportunities for health. The Colorado Community Engagement Alliance (CO-CEAL), funded by the National Institutes of Health (NIH), began with efforts to promote COVID-19 awareness and prevention and has since expanded to address chronic conditions and other health outcomes. CO-CEAL seeks to strengthen the translation of medical knowledge and scientific evidence into sustainable, culturally relevant practices and messages that resonate with communities. The initiative also aims to build capacity among community members and foster partnerships both between communities and the University and within communities themselves.

**Methods:**

Using the Transcreation Framework, CO-CEAL aims to engage with 5 communities in urban and rural Colorado to co-identify and co-address chronic disease-related issues with interventions that align with community cultural norms and values. CO-CEAL uses Group Model Building to identify leverage points in each community and uses the Community/Boot Camp Translation process to transcreate and disseminate messages and interventions related to chronic disease and mental health using a variety of mediums. CO-CEAL collaborates with community members, identified for their status as trusted messengers and having strong community connections, to build networks of community members from various sectors/roles to participate in project activities, including Boot Camp Translation, data collection, and participatory community meetings.

**Discussion:**

This study will provide an understanding of how to impact key drivers of disproportionate impacts on community co-identified health priorities by adapting and implementing appropriate interventions using community engagement strategies.

**Clinical trial registration:**

Identifier OT2HL158287.

## Introduction

### Background

The Colorado Community Engagement Alliance (formerly “Colorado Community Engagement Alliance against COVID-19”), or CO-CEAL, began in 2021 with funding as a CEAL Regional Team from the National Institutes of Health (NIH) in response to the COVID-19 pandemic. Initially, CO-CEAL aimed to maximize the reach of COVID-19 vaccines, testing, and treatments to Colorado’s most vulnerable populations. Over the past 3 years, CO-CEAL broadened its focus to address social determinants of health and related inequities disproportionately experienced by certain ethnic and racial communities; papers from this work have been published (1, 2). This change aligned with the shift in NIH’s focus and the health topics that were included in the study’s Common Survey (e.g., chronic disease, mental health, access to care, etc.).

This paper aims to describe the research protocol for CO-CEAL’s current multi-year study which began in April 2024. In this multi-year study, CO-CEAL uses Community-Based Participatory Research and the Transcreation Framework to ensure that materials and interventions are meaningful and sustainable. CO-CEAL leverages the Colorado Clinical and Translational Science Institute—Community Engagement infrastructure and other community-based and academic entities to extend reach, foster trust, and strengthen community-engaged research and community-driven solutions.

### Objectives

CO-CEAL’s mission is to address disproportionate impacts on health through active community engagement and outreach, capacity building, and long-lasting community partnerships to improve participation in health research. It seeks to understand how to translate evidence around effective disease prevention and intervention methods, as well as how to effectively transcreate related messaging and assess intervention uptake among participating communities. Specifically, our research focuses on the effective use of Group Model Building (GMB) and Community/Boot Camp Translation (BCT) (3) to identify factors influencing disproportionate impacts on health and designing or adapting interventions to address those factors to create change on an individual and community level. To this end, the CO-CEAL multi-year study aims to:

1. Identify systemic factors driving disproportionate impacts on health in participating CO-CEAL communities using Group Model Building.
2. Identify leverage points of intervention to positively influence the identified driving factors.
3. Design interventions to address the leverage points using Community/Boot Camp Translation.
4. Understand the extent to which interventions impact individual and community outcomes in each community using community surveys (e.g., Common Survey, Ecological Momentary Assessment) and interviews.

### Conceptual framework

The CO-CEAL multi-year study is grounded in the principles of Community Based Participatory Research (CBPR) (4) and systems science using GMB and BCT to inform and develop messages and interventions. CO-CEAL employs trusted community members, referred to as Community Connectors, to serve as on-the-ground field leads and data collectors and lead recruitment of study participants and community-based organizational partners.

In alignment with CBPR principles, CO-CEAL adapts and uses the Transcreation Framework for Community-engaged Behavioral Interventions to Reduce Health Disparities as the conceptual framework for our project (5). The Transcreation Framework was designed to provide a paradigm for designing and evaluating health interventions in real world settings using community engagement principles. Its use has been described in the setting of Spanish speaking Latinas with breast cancer (6, 7).

In Table 1, we display the Transcreation Framework’s seven steps along with our adaptations for our CO-CEAL multi-year study.

**Table 1:** Transcreation Framework with CO-CEAL multi-year study phases and adaptations.

Transcreation Framework steps	Colorado CEAL multi-year related study phase and adaptations
Identify community infrastructure and engage partners	Initial 6-month phase including Community Kickoff/Listening Sessions
Specify theory	Group Model Building phase—identify factors, with the community, contributing to disproportionate impacts on health and potential behavior change targets
Identify multiple inputs for new program	Group Model Building phase—Identify evidence-based interventions
Design intervention prototype	Boot Camp Translation phase—Transcreate interventions with community
Design study, methods and measures for community setting	Boot Camp Translation phase—Design implementation and relevant Ecological Momentary Assessment measures
Build community capacity for delivery	Implementation phase
Deliver “transcreated” intervention and monitor implementation processes	Implementation and Assessment phases

Transcreation has been a hallmark of our CO-CEAL work to date through our BCT process in years 1–3 where we engaged our partner communities to transcreate evidence about COVID-19 and COVID-19 vaccines and engaged them to disseminate the messaging (2). Using the BCT process, community teams learned the scientific evidence behind health topics (e.g., COVID-19, vaccinations, chronic diseases), identified key messages to communicate, and determined culturally appropriate ways to deliver those messages and convey the scientific information in a manner that resonated with community members. One example from the American Indian/Alaska Native (AI/AN) community was a “zine” with coloring pages, community member stories and testimonials, and characters created collaboratively with community members, depicting images and wording focused on protecting nature, community elders, and respecting sacred life (8). The Urban Black/African American community created a short YouTube video and a logo depicting the importance of protecting one’s family (9). Other communities created coloring books, bus shelter posters, stickers, water bottles, and duffle bags all with language, colors, and images that aligned with community values.

## Methods and analysis

### Project team

The CO-CEAL multi-year study has multi-Principal Investigator (PI) leadership of an academic PI (DN) and community PI (RGF). Co-Investigators bring expertise in the various methods used within the multi-year project. Individual staff members bring additional expertise in community engagement, project management, and reporting. Within each CO-CEAL partner community we have additionally contracted with community members, creating roles of Community Connectors.

The Community Connector model is an approach to community-based recruitment and retention that engages local members of the community, typically community leaders, as members of the research team and liaisons to the community members. While some Community Connectors may have experience as community health workers or *promotores de salud*, their role in this project is to support the research work rather than function as frontline public health professionals. The Community Connectors are recruited to the research team based on their unique skills and stature as respected community members with access to community gatherings, knowledge of local community groups and organizations, and established relationships with people throughout the community as well as more specific skills, such as cultural or language translation skills. Community Connectors have various roles and responsibilities, primarily as field leads (who serve as primary contacts for other community activities) and data collectors. They are compensated for their time and provided with relevant training for their roles, such as CITI Human Subjects Research training and data collection; they are also involved in poster and presentation development and dissemination, among other capacity-building opportunities. Community Connectors use their social and professional networks to identify and recruit participants for GMB and BCT community teams, surveys, and other project activities (e.g., community meetings, workshops, conferences). Community Connector teams become the primary point of contact for community members for the duration of the study.

### Setting

During our multi-year study, as in our original study period, CO-CEAL continues to work in five geographic and cultural communities throughout Colorado: the urban Latina/o/x community in Metro Denver and Pueblo, the rural Latina/o/x community in the San Luis Valley, the urban Black/African American community in Metro Denver, the rural East African immigrant community in Ft. Morgan and Greeley, and the American Indian/Alaska Native community along the Front Range and Metro Denver. These communities were initially chosen in 2021 due to experiencing chronic disease and COVID-19 at a disproportionate rate compared to other communities in Colorado. CO-CEAL partners with community members and individuals from community-based organizations, healthcare practices, and other relevant organizations for various project activities.

### Community engagement strategies

Community-engaged, participatory processes are central to the CO-CEAL multi-year study to ensure grass-roots community co-design, input and interpretation at each step. These processes are listed in Table 2 and described below. By collaborating with respected Community Connectors to invite community members and other partners to the table, we foster trust and build relationships between communities and the research team and reduce potential social desirability bias during engagement activities. Additionally, community participants, co-facilitators, co-facilitator assistants, and Advisory Group members are compensated based on activity (e.g., stipends, mileage, and hourly payments).

**Table 2:** Community engagement strategies.

Strategy	Description/purpose
Community Connectors	Build capacity for community-engaged research with community partners; various roles and functions
Community listening sessions	Ensure community input at key points during the project
Group Model Building	Engage community members in building a system map capturing factors that contribute to a selected health priority
Community/Boot Camp Translation	Community creation and development of an intervention and key messages to address priorities identified in Group Model Building
Community advisory group	Individuals from each participating community who provide insight and direction to overall project
Community-based data collection	Build capacity for community-engaged research with community partners and to ensure trusted reach to community members who might otherwise not be engaged
Community-guided data interpretation	Build capacity for community-engaged research with community partners and ensure interpretation of the data and conclusions are informed by the community

As of this writing, CO-CEAL has completed the first year of the multi-year study and finished community kick-offs/listening sessions and GMB. The team is completing baseline data collection and beginning BCT.

#### Community kick-off/listening sessions

The CO-CEAL multi-year study began by finalizing the health topic on which to focus for each of our partner communities via listening sessions and kick-off meetings. Sessions were held in person and virtually (e.g., Zoom) and carried out in English, Spanish (directly or with interpretation), and Somali based on the community’s preference and co-facilitated by the project multi-PI and Community Connectors. We held these sessions in each partner community with several purposes in mind. First, we returned important results from several of the prior Common Surveys conducted in each community. Common Surveys have been, and continue to be, collected by all CEAL Regional Teams. Prevalence of chronic illness was a main focus in Common Survey 3 and each community’s results were shared with them, respectively. We also shared our longitudinal analysis of Common Survey waves 1–3 which showed a significant impact on COVID vaccination rates through exposure to the BCT messaging. Second, we shared the plans for the various phases of our multi-year study with each community to increase interest and engagement, and gave individuals an opportunity to reflect on how they wished to participate in the various phases. Finally, using the Common Survey 3 results, data from the Colorado Equity Compass^1^ and health priority survey data from December 2023/January 2024, we confirmed with each community their chosen priority health topic of focus for the multi-year study.

Invitations to the kick-off/listening sessions were disseminated as broadly as possible with the support of our Community Connectors and prior BCT facilitators and participants, when appropriate. Word of mouth and social media were used to ensure as broad of representation as possible from each partner community.

#### Group Model Building

After the community kick-off/listening sessions, GMB (10–12) was carried out in each partner community to map and identify the key factors contributing to the priority health topic as well as leverage points to be developed into an intervention. GMB is a participatory process that brings together eight to ten individuals from various sectors of the community with lived experience to surface factors as well as the inter-relationships that contribute to a problem of interest. Over a series of meetings, workgroup participants discussed the origins, underlying causes, and possible solutions to a problem of interest through guided group activities. The process resulted in each community workgroup developing a visual diagram that showed how different factors are connected to the prioritized health topic and guided the collaborative identification of actionable areas to intervene through an impact and feasibility prioritization process. This process involved the GMB teams reviewing evidence for interventions when selecting a particular factor for intervention. Later in the project, representatives from all GMB workgroups will come together to prepare a policy brief that summarizes the health priorities and proposed areas for intervention, especially highlighting recommendations for addressing structural factors that contribute to disparities in chronic disease and communities’ ability to resolve them.

Community Connectors helped recruit prospective GMB participants and co-facilitators, which were finalized with support from the Community Engagement lead and GMB academic team. Most (four of five) community workgroups used a co-facilitation model that paired an academic team member with a community member and had a community co-facilitator assistant to provide support (e.g., meeting logistics, notes, etc.). One community workgroup was led by two community member co-facilitators who also shared the assistant duties. The community chose this approach due to cultural and linguistic preferences and norms, and consulted with the academic GMB team, as needed. GMB facilitation training occurred both before formal GMB sessions began and between the meetings to allow for adaptability and feedback during the process. Based on the community’s preference, GMB meetings were convened in a combination of in-person and virtual sessions or fully virtually. Duration of the GMB process in each community ranged from 5 to 6 months. To bolster engagement and be mindful of various factors, the research team, in collaboration with Community Connectors and GMB facilitation teams, considered the participants’ needs and concerns with regards to meeting timing and venues that would be accessible and safe, and prepared for and debriefed from meetings pertaining to sensitive topics.

#### Community/Boot Camp Translation

BCT constitutes the next phase of work in each partner community. The BCT groups will use the BCT process to devise or transcreate an existing intervention or interventions to address the factor(s) identified in the GMB process as key leverage points. These interventions could be similar to the community specific, culturally relevant informational messaging utilized successfully in our first three CEAL funding rounds, newly designed interventions, or they could be community specific, culturally relevant adaptations of existing evidence-based interventions.

In contrast to the BCT process used in our first three CEAL funding years, we will employ a traditional BCT process over 6–9 months rather than a rapid 8–10 week process (2). This longer BCT process will allow ample time for participants to learn about and refine the results of the GMB process and to design or adapt an existing intervention for their community. Participants in BCT function as research partners rather than research subjects and are the driving force behind the development of a product, message, and/or intervention that “works” for the intended users and communities. The BCT teams will also co-design the implementation and provide input into the Ecological Momentary Assessment (EMA) measures being used to evaluate the transcreated interventions.

The facilitation model for BCT will be similar to GMB, pairing an academic and community facilitator co-facilitation team and having a community co-facilitator assistant. BCT facilitation refresher training will be offered for the facilitation teams, along with longitudinal coaching of the teams. BCT co-facilitators and BCT assistants will be recruited in a variety of ways, including recommendations from Community Connectors and the Community Engagement lead, as well as, when suitable, re-engaging former BCT facilitators who continue to be a good fit for the role. BCT participants will be recruited in a collaborative manner with Community Connectors suggesting potential community partners to whom facilitation teams can reach out to invite. Additionally, former GMB participants may also be approached for participation. Each community will ideally have 12–14 community participants.

#### CO-CEAL advisory group

The CO-CEAL multi-year study includes an overarching advisory group to provide input from individuals from our communities and partner organizations that may not be intimately involved with aspects of the project. The advisory group will be comprised of 9 members representing key groups of influence including our 5 partner communities, the Trailhead Institute’s Regional Health Connectors, Rocky Mountain Prevention Research Center, The Care Collaborative (formerly PMCC/ECHO Colorado) and the Colorado Clinical and Translational Sciences Institute PACT Council. This group will meet quarterly with the multi-PIs and project staff to review and provide input on the project goals, operations and progress.

CO-CEAL’s various individual and organizational community partners and their roles are described in Table 3.

**Table 3:** CO-CEAL community partners and roles.

Key community partner name	Organization	Expertise, roles, and responsibilities with the team or study
Community Connector Field Leads	Various	Community field team leadership and project coordination support
Community Connector Data Collectors	Various	Survey recruitment and administration
Boot Camp Translation Community Participants	Various	Community expertise, material/messaging development and dissemination
Boot Camp Translation Community Facilitators	Various	Boot Camp Translation process co-facilitation
Group Model Building Community Participants	Various	Community expertise
Community Dissemination Partners	Various	Community expertise, material/messaging dissemination in communities
Group Model Building Community Facilitators	Various	Group Model Building process co-facilitation
CO-CEAL Advisors	Various	Broad expertise/influence across key communities and sectors

### Study design

The CO-CEAL multi-year study will primarily use a pre-post quasi-experimental design to assess the effectiveness of transcreated interventions for identified health priorities for each participating community. We will evaluate individual level uptake and exposure to the intervention or related materials and outcomes (e.g., behaviors, access to services, chronic disease prevalence, and social determinants of health) using EMA at baseline and post-intervention. We will use a secondary cohort design utilizing our Common Survey waves at baseline and post-intervention to gain an assessment of effectiveness using a broader population in each community that will include exposed and non-exposed individuals. CO-CEAL will also carry out evaluations of the GMB and BCT processes via interviews with participants to understand their experiences and identify areas of improvement.

### Study population

Project and study participants will live or work in the aforementioned CO-CEAL partner communities. Study participants (i.e., EMA and Common Survey participants) will include adults ages 18 or above, those who can read and write English or Spanish, and Somali speaking and reading adults in the rural East African immigrant community. Community Connectors, GMB and BCT facilitators, assistants, and community participants are considered part of the project/research team and will not be eligible as study participants. It is anticipated that female and male participants will participate in the study in the same proportions as they are represented in the target communities in the research. Likewise, individuals of various races/ethnicities (Black/African American, East African immigrant, Latina/o/x or Hispanic, American Indian/Alaska Native) will participate as they are represented in the community. Those who do not meet inclusion criteria will be excluded from study activities.

### Intervention and comparator

Each community will create or adapt an existing intervention that will address their community’s chosen health priority. Communities will use the GMB process to identify factors that contribute to the health concern upon which communities can take action, develop a system map, and develop, transcreate, and refine their intervention during BCT. Our primary comparator will be exposure to the intervention in each community. Exposure will be primarily assessed via EMA pre- and post-intervention using a recruited set of community members. Additionally, we will intentionally recruit a sub-set of Common Survey participants who are not part of the EMA evaluation to permit a cohort fashion exposed vs. not exposed comparison.

### Recruitment and enrollment

As this is a pre-post design, CO-CEAL will work with the same participants for both the baseline and post-intervention EMA and Common Survey administration. Individuals may participate in one or both evaluation activities, depending on a community’s interests and capacity. We are confident that we will have high participant retention, as our Community Connector data collectors are respected individuals in their communities and foster trust with participants. Over the past 3 years of the study, we have had a retention rate of over 80% for survey participants and all interested Community Connectors and other community partners have stayed.

#### Ecological Momentary Assessment

CO-CEAL plans to recruit 500 respondents across all participating communities, with sample sizes relative to the community’s size. EMA respondents will be recruited via Community Connector data collectors, as CO-CEAL has successfully done for past Common Surveys. Potential participants will be given information about the project (e.g., in person or via social media) and then complete an initial eligibility survey, followed by an online consent if they are eligible. Reloadable gift cards will be used to compensate EMA respondents $5 for each one-to-two-minute survey completed over a 1 week period. This dollar amount was determined given the brevity of the survey and aligns with our former Health Knowledge Monitoring and Response System (HKMRS) pilot project that had a similar survey structure (iHeard Colorado^2^). Power calculations are based on anticipated change in outcomes/behaviors both aggregated across all communities and for individual communities, as well.

#### Common Survey

Common Survey participants will be recruited as we have done in prior waves, utilizing Community Connector Data Collectors who have deep connections into our partner communities with known, trusted relationships. As in past waves, CO-CEAL anticipates recruiting 1,000 respondents for the first wave during the multi-year study and then returning to these same respondents in the second wave in a cohort approach. Participants will be compensated $50 per survey.

#### Qualitative

With input from the Community Connector teams, the research team will contact up to 25 facilitators and participants (approximately five from each community) from GMB and BCT to participate in individual interviews to understand their experiences and gain an in-depth perception of the group processes. We chose this sample size to ensure representation from a variety of roles and community perspectives and to achieve thematic saturation. We will interview participants until thematic saturation is reached (13, 14). Participants will be compensated $40 per interview.

### Data collection approach and measures

#### Ecological Momentary Assessment

EMA/mHealth technology tracks behavior across time and context, which allows for identifying momentary factors that may be associated with key outcomes. In the current study, we will employ EMA to assess the uptake and impacts of our interventions on communities at the individual level. EMA is a survey-based technique that is distributed via mobile, phone-based surveying. We have developed experience with weekly, mobile, phone-based surveying in iHeard Colorado. Based on EMA best practice (15), we will use signal contingent (15–17), EMA messaging that utilizes text messages with short surveys sent randomly throughout the day that participants respond to allowing for capturing individual behaviors, feelings, attitudes, and contextual factors (e.g., accessibility) as they unfold across time and context. We will assess key outcomes related to behaviors, access, etc. based on the community identified intervention targets from our GMBs and BCTs in each community. According to best practice, the EMA observation period for each participant will be 7 days, with 4–5 text messages sent throughout the day. This will allow for identifying patterns within and across day of participant outcomes of interest to the intervention. These assessments will be collected, as appropriate to the intervention target, up to daily during defined weeks during Years 2 and 4, prior, potentially during, and post intervention implementation and dissemination.

#### Common Survey

We will add similar, yet static measures from the EMA in each community to the Common Survey to assess exposure and changes in response to the interventions. Items to assess exposure to the interventions in each community will assess message awareness and cultural adaptation effects from the interventions designed in BCT. In prior Common Survey waves, we successfully used images of the transcreated intervention materials in each community to assess exposure. Exposure and awareness will be assessed via one adapted item which asks, “Have you recently seen a [priority topic] message on [distribution channel(s)] that shows [brief message description]?” with responses of, “Yes, definitely; Yes, probably; No; Do not know.” Additionally, the communication materials developed by the community will be evaluated for effectiveness using items related to knowledge and attitudes about the chosen health priority. We will also assess cultural adaptation through participants’ rating of the communication materials including perceptions of trustworthiness, personal relevance, personalization, cultural fit, appearance, and information—using a previously developed measure of cultural sensitivity. Items will be adapted from Singelis and colleagues’ measure of their two-dimensional theory of cultural sensitivity in health communication (18). The initial Common Survey wave will be administered prior to the intervention with items relevant to communities’ priority health topics and anticipated transcreated interventions in each community. The follow-up Common Survey will be administered in Year 4.

#### Qualitative

CO-CEAL will conduct community key informant interviews, gather field notes and hold debrief conversations with participating community team members from GMB and BCT. These efforts will offer insight into participants’ experiences in the processes and identify areas of improvement. Interviews will also help us understand the fidelity of the GMB and BCT processes and the level of cross-sector collaborations and community capacity building that was fostered.

### Data management

#### Quantitative

CO-CEAL utilizes an NIH-provided survey called the Common Survey for the recurring pre/post standardized community surveys. CO-CEAL includes additional questions relevant to the intervention’s health topic and created materials. For the multi-year study, CO-CEAL will use Common Survey 4. Surveys will be administered by Qualtrics secure online survey system, which can be taken via mobile application on a phone, tablet, or computer; if not Qualtrics, EMA surveys will be administered via another secure platform. Identifying information will be collected to ensure ability to retain participants and conduct follow-up surveys; however, once collected identifiable information and survey response data will be stored separately. Surveys completed online via Qualtrics remain confidential and secure and will be accessible via login and password to research team members. Common Surveys may be conducted in person, by mail, online/video call, or phone with a Community Connector data collector while EMA surveys will use a text/SMS or similar platform.

Survey participants will be consented electronically in Qualtrics. Postcard consent will be obtained even with face-to-face with assistance from a member of the study team. If requested, participants will receive a written consent document and additional verbal consent will be assessed. Completion of the survey will then indicate consent.

If a Community Connector data collector collects survey response data by phone or video call, they will first verify the identity of the respondent and then enter their responses directly into the Qualtrics system to ensure the security and confidentiality of participants. Mailed surveys will be returned directly to the University of Colorado. No identifying information will be included in mailed surveys, only a study ID. For all aspects of the study, only members of the research team, including Research Associates, analyst, and Principal Investigators will have access to subjects’ identities/identifiers and audio-recordings. All such staff members will be asked to sign confidentiality agreements, agreeing not to reveal the names or other identifiers of study participants to anyone outside the research team. All personal contact information will be erased/destroyed once their participation in the research is completed.

#### Qualitative

Interview participants will be consented verbally and provided with a postcard consent. Interviews will be conducted via phone or virtual platform (e.g., Zoom). All interview data will be stored on a secure server. Audio recordings will be kept on the secure server until a transcript is created. Transcripts will be redacted of all identifiable information. Once a transcript is created, the recording will be deleted. Identifiable interview data will be kept confidential and will only be accessible to the study team. Deidentified interview data, in the form of redacted transcripts, will be shared with funders via a secure web-based platform.

All data will be synthesized for reporting purposes and no identifiable information will be shared in any reports. All members of the research team will keep all identifiable survey data confidential within the research team.

### Quantitative data analysis

#### Ecological Momentary Assessment

Though EMA uses longitudinal data trends, it is reasonable to assume the completeness and fidelity of those data may include a substantial amount of missingness (e.g., individuals may miss certain EMA data entry points), particularly in non-clinically controlled settings. Because of this, we use a more conservative F-test (via a mixed-ANOVA) for power calculations to account for the potential of substantial within-person missingness which allows us to compare both between groups (one community compared to another) within groups (change over time within a single community) and potential interactions.

Assuming 95% power, at least four time points, an intraclass correlation of 0.4 (also a conservative estimate), and six groups with unequal sample sizes (our latest sample sizes range from the smallest group of consisting of 4% to the largest group comprising 27%), we estimate that 426 individuals are needed to detect differences using a small effect size (Cohen’s d = 0.1). Using a final sample of 500 individuals allows for an approximate 15% attrition rate (a recent meta-analysis on 30 EMA studies estimated an average attrition rate of 17%) (19). We believe these calculations well insulate our study from potential missingness, attrition, and type II errors.

With respect to individual community level analysis, the above power calculation will also allow us to conduct a within groups investigation by community (e.g., detecting changes, relative to the outcome, within a single community). Given the smallest community in our previous work comprised 4%, we anticipate that given a target sample size of 500 will produce at least 20 individuals in our urban AI/AN community. Using the following assumptions of 95% power, intraclass correlations of 0.4, and 7 longitudinal data points (assuming we get at least one EMA data point per day), we would need 21 individuals in order to detect changes with a medium effect size (Cohen’s *f* = 0.3). While this gives us the minimum number of individuals for the smallest group, this also allows for more complex analyses with smaller effect sizes for the larger groups. For example, our largest group in our previous study comprised 27% of the sample, equating to a sample size of 135 in our 500 target sample. Given the same power calculation assumptions with the aforementioned AI/AN community, a sample of 135 would not only allow us to detect changes with a much smaller effect size (Cohen’s *f* = 0.12), but would also allow for more robust statistical testing for sensitivity analyses using covariates (e.g., age, education, income) and stratifications (e.g., changes among females, changes among those in certain age demographics, changes among those with certain health conditions).

#### Ecological Momentary Assessment analytic plan

EMA data presents complexities because of its nested structure: assessments (level 1) are nested within days (level 2), which are in turn nested within individuals (level 3). Given this nature, we plan to use the method described by Ma and colleagues which implements a linear- mixed effects model (LMM) with random effects at each level (20). This model analyzes both within-subject effects (e.g., longitudinal trends in an individual) and between-subject effects (e.g., community level comparisons). Both pre-intervention and post-intervention data will be used to analyze trends (e.g., slopes) in EMA measures across time. LMMs can also account for covariates within the model and are level specific (e.g., sex/age for level 1, day of the week, such as a weekend for level 2, etc.). All outcomes will be tested for normality, with adjustments made to skewed data (e.g., log transformations). Statistical significance will be assessed at the 0.05 level.

While LMMs will be used to estimate within- and between-subject trends in EMA outcomes, we will also employ interrupted time-series analysis to complement these models. Interrupted time-series is particularly suited for evaluating the impact of an intervention on outcomes over time by modeling level and slope changes between pre- and post-intervention periods (21). This approach is consistent with other EMA protocols in which interrupted time-series has been used to estimate causal effects in real-world, longitudinal settings. Lastly, beyond analyzing longitudinal trends post-intervention, we will also correlate EMA measures with common survey items to assess how changes in EMA trends are associated with social determinants of health, attitudes, and trust as well as exposure to any community level intervention. This can be done using the same LMM framework as described previously.

#### Outcomes

Given that communities will have the flexibility to choose tailored outcomes based on Common Survey 3, the above analyses will allow for flexibility in testing research questions given those chosen outcomes. For example, if a given community chooses a mental health indicator (e.g., the GAD-7 scale which assesses general anxiety disorder), the proposed analyses will allow for the testing of several research questions: (1) Are there changes/variability in anxiety levels in the week of data collection during the first assessment of EMA? (2) Are there changes/variability in anxiety levels in the week of data collection, post-intervention, during the second assessment of EMA? And (3) Is there a significant change in these slopes of anxiety, comparatively, from each EMA data assessment period (i.e., did the intervention between EMA periods significantly change the slopes of anxiety when comparing EMA1 to EMA2)? Other outcomes, depending on what individual communities choose, can be modified to fit these research questions to assess statistically significant changes.

#### Common Survey

As noted above, we will add equivalent measures from the EMA in each community to the Common Survey to assess changes in response to the interventions. Additionally, we will add items to assess exposure to the interventions in each community. In prior Common Survey waves, we successfully used images of the transcreated intervention materials in each community to assess exposure. The initial Common Survey baseline wave will be administered prior to the interventions and include items relevant to the priority health topics and anticipated transcreated interventions in each community.

The domains for the Common Survey include demographics, social determinants of health, information and trust. Aside from general descriptive statistics, we will conduct bivariate analyses comparing subgroup distributions (e.g., by language, community) to overall sample proportions or means, using chi-square or t-tests as appropriate. We will also create a data dashboard(s) to illustrate data on health behaviors, attitudes, and exposure to intervention materials.

We will conduct linear/logistic regression analyses (depending on the outcome) to explore the relationships between social determinants of health, information and trust and exposure to intervention materials on chronic disease outcomes. In our prior Common Survey samples we have seen sufficient power to observe significant odds ratios for health behavior differences across communities in vaccine uptake, as well as impacts of trust on vaccine behaviors. With an anticipated sample size that is similar in our future Common Survey samples we believe we will have sufficient power to observe pre-post differences in health behaviors from years 1–4.

### Qualitative data analysis

The CO-CEAL qualitative expert (DF) will lead the GMB and BCT interview analysis with the assistance of a research services professional with qualitative experience (MW). We will analyze interview data using inductive coding methods to identify themes. The analysis will use a grounded hermeneutic editing approach to help identify themes that are “grounded” or developed from an interpretation of the data (22). Emerging themes will be shared with study team members, including GMB and BCT leads, for comments and questions before the analysts return to the data for further coding and review. Case-based matrices will be used iteratively with the study team to further organize the coding schemas into refined categories and to help identify common themes and patterns in the data, plus contradictory and unique cases or findings (23). We will use data analysis software (e.g., ATLAS.ti) to facilitate this process.

#### Implementation timeline

**Figure 1:**
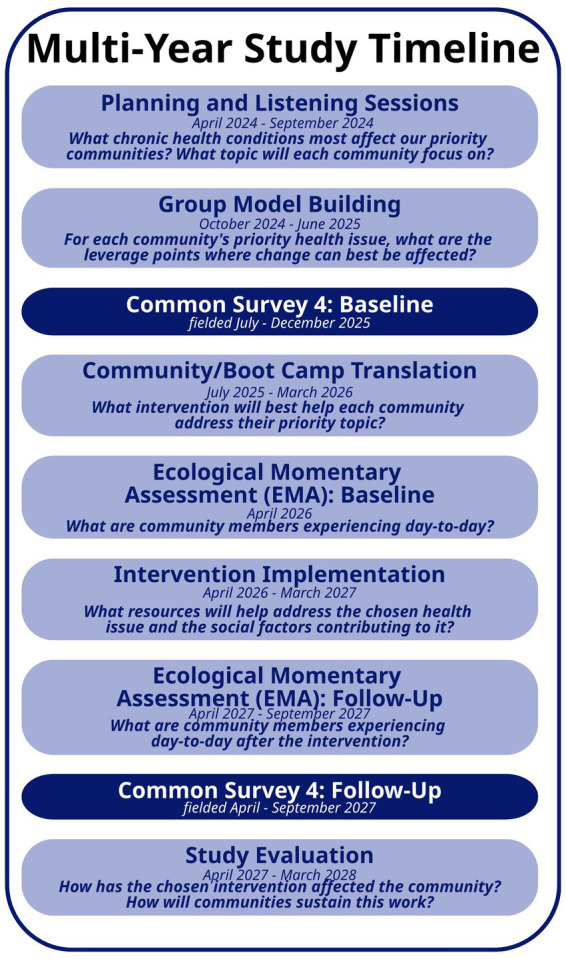
Multi-year study timeline.

The CO-CEAL multi-year study spans 4 years (Figure 1). The first year focuses on confirming health priority topics and project activities to set the stage for the remainder of the project. GMB spans the first 2 years to inform the intervention plans, followed by BCT to develop and/or modify existing interventions to address the health topics and leverage points identified in earlier stages. Surveys will be administered in year 2 (Common Survey) and early in year 3 (EMA) prior to interventions beginning. Community interventions will be carried out in year 3, followed by post-intervention surveys, data analysis, and other evaluation activities in year 4.

## Discussion

### Summary

CO-CEAL’s multi-year study represents an ambitious effort to impact community co-identified health needs in five different Colorado communities using common methods based on the Transcreation Framework. Each community will be engaged as partners to define their health topic, create a systems-based model of the factors which impact that health issue through GMB, develop an intervention to impact key leverage points in the model through BCT, and assess the outcomes through EMA and the Common Surveys. In the spirit of co-learning and co-creation inherent in CBPR we anticipate substantial mutual learning through the implementation of this study.

### Strengths and limitations

CO-CEAL is a community driven project that relies heavily on community members’ authentic engagement and participation. This is one of the project’s greatest strengths, as health priority topics are chosen by members of the communities in which the interventions are implemented, and materials and messages created are culturally and linguistically appropriate for the intended audiences.

A potential limitation on participation is competing priorities for community members’ time which could create a selection bias favoring those with more time available and could create delays in the project timeline. Most community members with whom we engage, including Community Connectors and project participants, have at least one job and many have families, thus time for CO-CEAL activities is somewhat limited. We strive to be inclusive of those who have multiple responsibilities, while recognizing that this may extend the time needed for project participation, resulting in delays in implementation at various points. To anticipate these constraints, we have built places into our timeline where we will be able to recover and catch up to ensure completion by the end of our fourth year.

## Ethics and dissemination

### Ethical considerations and declarations

This study has been reviewed and approved by the primary study site human subjects review board, Colorado Multiple Institutional Review Board (COMIRB) as “exempt.” COMIRB approved a waiver of documentation of consent, given that participant identifiers will not be stored along with study response data. Notification of withdrawal of participation will be given to the study team or Community Connector data collectors. Protocol modifications are submitted for approval to COMIRB prior to implementation. The principal investigators declare no financial conflicts or other competing interests.

### Dissemination plans

Project findings will be disseminated in a variety of both community and academic venues. CO-CEAL hosts an annual statewide conference in collaboration with two other University entities that includes CO-CEAL community partners, community-based organizations, local public health department personnel, and primary care practice staff. This conference often features a poster session, oral presentations and panels, and skill-building workshops focused on CO-CEAL research findings and practices; community partners and CO-CEAL participants often co-author and participate in many of these activities. CO-CEAL also presents findings at other statewide and national primary care and public health-focused conferences. Throughout the project, CO-CEAL releases quarterly project newsletters to inform community and academic partners of the progress of our work. Additionally, CO-CEAL has and will continue to submit conference posters and presentation abstracts and manuscripts to academic journals for publication, many of which authentically engage community participants as co-authors. As of writing, CO-CEAL has published a brief report and one manuscript, was a co-author on another manuscript, and has other manuscripts in process.

### Conclusion

CO-CEAL aims to advance understanding of how to address factors that influence disproportionate impacts on health in Colorado communities experiencing the greatest burden of disease, and how to adapt and implement stakeholder engagement and capacity-building methods to achieve these goals. It will also expand knowledge on how to use digital health technology (i.e., EMA) as part of community-engaged approaches and translate evidence and implement effective interventions for chronic diseases and other contributors to differences in health outcomes.

## Footnotes

1
https://coequitycompass.org

2
https://co.iheard.org
